# Populus Candicans

**Published:** 1888-05

**Authors:** C. F. Nichols


### POPULUS CANDICANS.

About 1875 I had two provers of Populus candicans (known about here as "balm of Gilead," but only resembling the charmed Oriental Mecca-balsam or Gilead-balm. Peanut oil resembles the oil of the olive, and hence is used to adulterate. However, *the resinous gum exuding from buds and stalks* of this well-known tree, deliciously aromatic in perfume, is widely used to heal wounds, open sores and eruptions, and often suppresses these latter to the harm of the patient. It should be understood that most persons apply this gum without noticeable poisoning, yet, like Arnica, it is dangerous. (Perhaps the poison comes from insects, as thought by Dr. Hering. Gnats and flies are abundant prisoners sticking to the buds). I have seen many poisonings of poison ivy and oak, but never saw blisters of *such size* as in S. E. C.'s proving. The size, with shape hanging down like water bags, is peculiar, larger than from Crot-tig.

1. Dr. Haworth, Andover, Mass., a very careful observer, took tinct. and 30c. several times about ten years (1870–82) and noted several poisonings. He did not distinguish between internal and external (accidental) provings.

2. Miss S. E. C., a patient of mine, dark, spare, wiry, in good health, about thirty-five, teacher, working hard at teaching until she was poisoned when preparing the freshly gathered buds for domestic use in Alcohol, April, 1875.

Eruptions. Five hours after she had burning, prickling on face, chest, and hands, parts became dark red and swollen, small vesicles appeared within twelve hours, directly increasing in size and commingling with *watery, acrid,* sticky oozing. The blisters remarkable in size and shape, *hanging down* like bags of water, as large as walnuts. S. E. C.

*Sensation of eruption,* about to break out (for years.) S. E. C., Haworth.

*Sensation of perspiration,* about to break out, but sweat rarely occurred, skin harsh and dry. S. E. C.

*Cold-sores* on lips, tingling, burning with *acrid watery* oozing, (never turning to pus, but drying over). Vertigo and vanishing of thought until hydroa appeared. (Haworth, two cases reported.) Sores and raw spots in various places. (Haw.) The whole surface of body, especially back and abdomen, remained *for months so lacking sensibility that vigorous rubbing and pounding by a strongly muscular woman were borne without pain, were almost unfelt,* but craved by the patient, who *feared paralysis.* (S. E. C.) Patient also craved rubbing to relieve chilly sensation internal and external. (S. E. C.)

Chilly, yet with burning stinging below surface, chest and abdomen. (S. E. C.)

Skin generally dry, harsh, lacking perspiration. (S. E. C.) Fine papillary eruption on face and trunk appeared annually (April), growing less abundant (under Rhus) ten years. (S. E. C.)

Mental, etc. Apprehensive; expects death and fears it; loquacious, discussing *repeatedly her symptoms with every one who calls;* irresolute, irritable. (S. E. C.)

Vanishing of thought. (S. E. C., Haw., two cases.)

Vertigo: as if from sunstroke (with pallid face), worse when lifting head, from motion. (Haw.)

Dreams frightful, vivid (Haw.) S. E. C., always of dead people.

Head. Sawing pain through left temple; sensation of weight in vertex; hot head suddenly, with numb, cold extremities; sensation as if left eye were twisted (during the severe headache). (Head hot, Haw.)

Face yellow. (S. E. C.)

Tongue feels thick and numb; speech thick; white, dry tongue. (S. E. C.)

No appetite, loathes meat, bitter taste, sweet taste. Vomits bile A. M. (S. E. C.) Vomits frequently, bitter. (Haw.) Nausea with sinking at epigastrium. (S. E. C.) Flatus with colic, doubles forward. Pain and general enlargement in right hypochond. (S. E. C.)

Diarrhœa (green, watery stools) alternates with constipation. (S. E. C.)

Wears her garments loose (abdomen and chest. S. E. C.).

Urine increased, red, dark, light, phosphates abundant (no albumen). (S. E. C.) Many phosphates, very frequent urination at night. (Haw.)

Menses scanty, a year (1875). *Absent* 1876. (S. E. C.) Became copious (while health was improving). Early with dysmenorrhœa, relieved by hot cloths, 1877–78. (S. E. C.) All symptoms worse before menses. (S. E. C.)

Vaginal burning, as if scalded.

But little leucorrhœa. Very persistent for years. (S. E. C.)

Burning of mucous membranes of *throat*, nostrils, and *vagina*, very distressing in throat, as if from swallowing hot fat. (Haw., two cases.)

Dry, asthmatic breathing, dyspnœa. *Sits bent forward* with dry cough. *Worse* (breathing) *when lifting arms.* (S. E. C.) Dull cardiac pain, fine stitches, palpitation with vertigo when rising or lying on left side. (Her brother has valvular disease, following rheumatism.) She has not previously discovered heart symptoms. For a year the heart sounds were irregular, usually muffled, with systolic murmur, especially noticed *before menstruation.* Pulse slow, about sixty (her normal pulse seventy-two). (S. E. C.)

Back numb throughout its whole length, as if numbness radiated from the spine, (associated with the abdominal loss of sensation). (S. E. C.)

Finger ends thickened, horny, insensible to pricking or pinching, nails not thickened. (S. E. C.)

Finger nails occasionally blue, as in ague. (S. E. C.)

Sleepless after midnight, usually until noon. (S. E. C.) Of case S. E. C., my patient was given numerous remedies. Rhus proved more serviceable than others seemingly well indicated. She is now in fair health.

If *Guiding Symptoms* want to epitomize some more radical nosodes, I have had clinical experience with Psoria, Medorrhine, Syphilinum, Tubercul., and Alb.

Perhaps some of my notes on Piper Methysticum at Honolulu might be helpful.

C. F. NICHOLS.

